# The Role of Antibodies and Their Receptors in Protection Against Ordered Protein Assembly in Neurodegeneration

**DOI:** 10.3389/fimmu.2019.01139

**Published:** 2019-05-31

**Authors:** Taxiarchis Katsinelos, Benjamin J. Tuck, Aamir S. Mukadam, William A. McEwan

**Affiliations:** Department of Clinical Neurosciences, UK Dementia Research Institute at the University of Cambridge, Cambridge, United Kingdom

**Keywords:** prion-like proteins, neurodegeneration, tau (MAPT), Fc receptor, microglia, antibody immunity, alpha-synuclein, beta-amyloid

## Abstract

Ordered assemblies of proteins are found in the postmortem brains of sufferers of several neurodegenerative diseases. The cytoplasmic microtubule associated protein tau and alpha-synuclein (αS) are found in an assembled state in Alzheimer's disease and Parkinson's disease, respectively. An accumulating body of evidence suggests a “prion-like” mechanism of spread of these assemblies through the diseased brain. Under this hypothesis, assembled variants of these proteins promote the conversion of native proteins to the assembled state. This likely inflicts pathology on cells of the brain through a toxic gain-of-function mechanism. Experiments in animal models of tau and αS pathology have demonstrated that the passive transfer of anti-tau or anti-αS antibodies induces a reduction in the levels of assembled proteins. This is further accompanied by improvements in neurological function and preservation of brain volume. Immunotherapy is therefore considered one of the brightest hopes as a therapeutic avenue in an area currently without disease-modifying therapy. Following a series of disappointing clinical trials targeting beta-amyloid, a peptide that accumulates in the extracellular spaces of the AD brain, attention is turning to active and passive immunotherapies that target tau and αS. However, there are several remaining uncertainties concerning the mechanism by which antibodies afford protection against self-propagating protein conformations. This review will discuss current understanding of how antibodies and their receptors can be brought to bear on proteins involved in neurodegeneration. Parallels will be made to antibody-mediated protection against classical viral infections. Common mechanisms that may contribute to protection against self-propagating protein conformations include blocking the entry of protein “seeds” to cells, clearance of immune complexes by microglia, and the intracellular protein degradation pathway initiated by cytoplasmic antibodies via the Fc receptor TRIM21. As with anti-viral immunity, protective mechanisms may be accompanied by the activation of immune signaling pathways and we will discuss the suitability of such activation in the neurological setting.

## Proteopathy in Neurodegeneration

Following the death of his patient, Auguste Deter, in 1906, Alois Alzheimer described the presence of abundant extracellular plaques and intracellular neurofibrillary tangles in her brain (1). These lesions were subsequently shown to be widely distributed in the brains of sufferers of the disease that went on to take Alzheimer's name. The plaques and tangles are now known to comprise of assemblies of the proteins amyloid-β (Aβ) and hyperphosphorylated microtubule associated tau, respectively. Alzheimer's disease (AD) is the most common of a heterogeneous family of age-related neurodegenerative disorders characterized by the deposition of specific protein assemblies in the brain. This includes progressive supranuclear palsy (PSP), corticobasal degeneration and Pick's disease, where tau deposition is observed; dementia with Lewy bodies and Parkinson's disease (PD) where cytoplasmic protein α-synuclein (αS) deposits are observed; sporadic Creutzfeldt-Jakob disease, where the membrane-anchored prion protein, PrP, is deposited and, finally, amyotrophic lateral sclerosis where TAR DNA binding protein 43 (TDP-43) is implicated. The common characteristics of the protein assemblies among these pathological conditions is that they exhibit an ordered fibrillar structure, known as amyloid, as well as a range of smaller assemblies generally referred to as oligomers. Together, the age-related neurodegenerative diseases are one of the most pressing biomedical and societal problems. Dementia, of which AD is the most common cause, affects around 50 million people worldwide and numbers are expected to double before the middle of the 21st century. Critically, there are currently no treatments that slow or prevent the progression of any of the age-related neurodegenerative diseases.

Findings over the past few decades place protein aggregation as a central mediator of pathology. Human genetics has revealed numerous mutations in the genes that encode the aggregating proteins themselves. A suite of more than 40 mutations in tau cause inherited dementias, with evidence of tau fibrils in brain tissue (2). Mutations in αS lead to inherited forms of Parkinson's disease and, in certain cases, an acceleration of *in vitro* αS fibrilization (3, 4). Mutations in the gene that encodes amyloid precursor protein (APP), the protein from which the Aβ peptide is derived, lead to increased levels of the aggregation-prone Aβ_42_ and familial AD (5). Other mutations in genes responsible for processing these proteins, such as the proteases responsible for the generation of Aβ, or in clearing misfolded proteins species, such as the AAA ATPase p97/VCP, can also lead to inherited variants of neurodegenerative diseases (6, 7). Collectively, these genetic associations suggest that the accumulation of protein aggregates causes neurodegeneration. For AD, the prevailing framework of disease progression is the amyloid cascade hypothesis (8, 9). Under this hypothesis, the accumulation of Aβ plaques drives pathological consequences that include the formation of tau fibrils and neuronal cell death. Therapeutic approaches in AD have therefore focused on preventing the production of Aβ, or promoting its clearance. A series of disappointing, high profile clinical trials have led to the critical reappraisal and amendment of the amyloid cascade hypothesis, or to propose earlier intervention, since the downstream events unleashed by Aβ accumulation may be irreversible (5, 10). Therapeutic approaches that target tau in AD are therefore considered promising routes for future intervention. Of the 20 therapeutic strategies that target tau that have reached clinical, nine are based on passive transfer or eliciting of antibodies (11). A further two therapies that target αS have also reached the clinic. Immunotherapy therefore represents one of the brightest hopes for modifying disease progression in age-related dementias.

## Protein Assemblies as Propagating Endo-Pathogens

**Figure 1:**
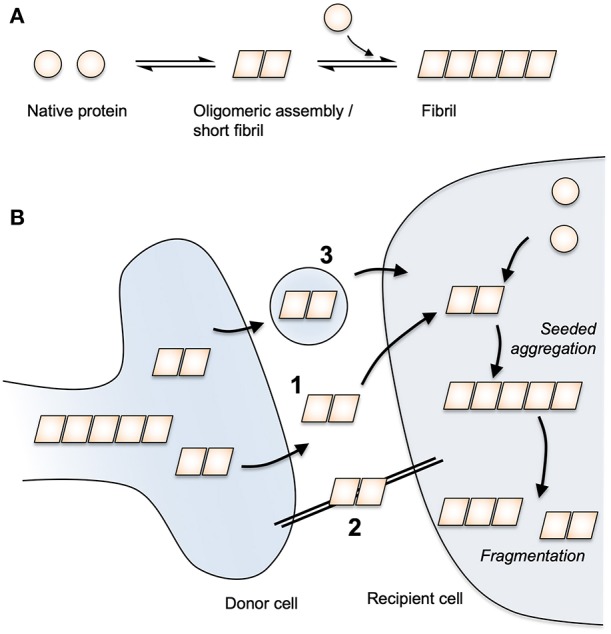
Protein aggregation and prion-like spread. **(A)** Native protein undergoes a spontaneous conversion to an assembled state. Assemblies above a critical size are able to extend via the addition of native protein monomers to form a fibril. **(B)** Assembled protein species are able to transmit between cells via routes that may include (1) free protein release and uptake, (2) tunneling nanotubes and (3) extracellular vesicles. Once taken up to the cytoplasm of a neighbouring cell, seeded aggregation occurs through the templated addition of native protein. By fragmenting, fibrils can exponentially amplify in number.

The occurrence of protein deposits was long considered a cell-autonomous feature of neurodegeneration. Over the past few decades, this view has been challenged by a body of research demonstrating that pathological protein conformations can provoke native protein to adopt the assembled form. By consuming pools of native cellular proteins, the assembled variants can sustain their propagation through time and space within the affected brain. The prototypic example of this behavior is the prion protein, PrP, wherein the normal cellular variant, PrP^C^, is converted to a pathogenic variant, PrP^Sc^. Most cases of prion disease are sporadic or inherited though, in rare cases, disease can be acquired from the environment by eating diseased meat or human brain as occurred in now-abandoned tribal rituals. The model of templated protein aggregation was proposed as a common mechanism in neurodegeneration when it was shown that Aβ could be induced to aggregate in mice expressing APP (12, 13). As an extracellular peptide, the seeded aggregation of Aβ likely relies on direct contact between introduced seed and the available pools of peptide. For other proteins such as tau and αS, which are expressed in the cytoplasm, pools of native protein are maintained within cell-limiting membranes, thereby limiting contact between seed and substrate. Seeded aggregation of cytoplasmic proteins was nonetheless demonstrated when AD brain homogenate was found to induce tau pathology in mice expressing wild-type human tau (14). In cultured cell systems, protein misfolding could be transmitted from the extracellular environment to cytoplasmic tau pools (15). Similar properties have been demonstrated for αS, TDP-43, and huntingtin, the protein whose expanded polyglutamine tract is implicated in Huntington's disease (16–19). Thus, although diverse in their clinical manifestations, it is possible that age-related neurodegenerative diseases share a common “prion-like” mechanism of dissemination though affected brains (20) (Figure 1).

Understanding the molecular mechanisms governing the transfer of pathology between cells is central to any mechanism-based intervention. For immunotherapy against tau and αS, the issue is key as it determines which pool of protein should preferentially be targeted. *In vitro* studies in neurons demonstrate that tau misfolding can be transferred across synapses (21, 22). This is consistent with animal work, which suggests that tau pathology is preferentially transmitted between connected regions of the brain (23–25). Furthermore, imaging of human brains using positron emission tomography (PET) tracers reveals that network connectivity is correlated with tau pathology, consistent with transfer of tau misfolding along synaptically connected pathways in the brain (26). Extracellular naked protein assemblies transiting between neurons thus represent an attractive target for immunotherapy as they are physically accessible to antibodies. However, other mechanisms of intercellular transfer have been described. For instance, exosomes and extracellular vesicles can contain tau and transmit pathology (27, 28) and tunneling nanotubes, actin-containing structures that bridge cells, can transmit pathology in culture (29, 30). There also remains discussion around the contribution of prion-like spread vs. cell-autonomous aggregation (31). Assuming that protein seeds are not obtained from the environment, then cell-autonomous aggregation must, at least, be responsible for the generation of the original seed. In this way, the extent to which pathology is governed by cell autonomous vs. prion-like mechanisms, and the route of such spread, define the limits of what any given therapeutic approach can achieve.

## Antibodies in the Brain

**Figure 2:**
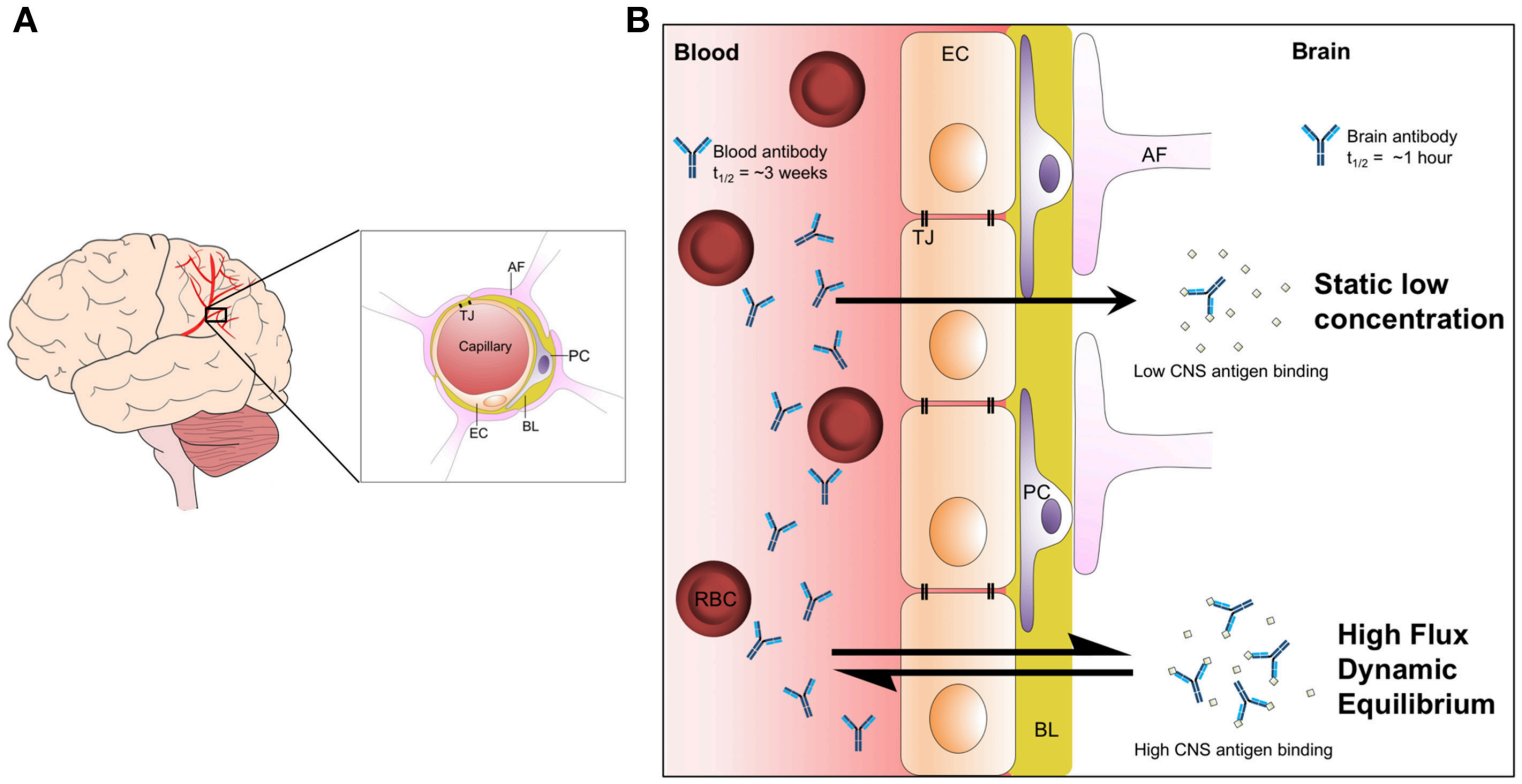
The maintenance of brain antibody levels. **(A)** Graphical representation of blood vessels in the brain and the cellular structure of the BBB. Endothelial cells in blood vessels interact via tight junctions, restricting the passage of solutes to the CNS. Pericytes bind to the basal lamina and provide structural support to the barrier. Astrocytic foot processes extend from the interstitial spaces to interact with the basal lamina and surrounding cells. **(B)** Two models of antibody cycling into the CNS. Under a model of static, low concentration in the CNS, antigen binding is highly restricted. However, a model where antibodies rapidly cycle in and out of the brain permits continuous bathing of brain antigens in dilute antibody solution. Over time, this model allows much higher levels of antigen binding. Evidence in support of such a model includes the observation that antibody half-life in the brain is <1 h, compared to around 3 weeks in serum. AF, astrocyte foot; BL, basal lamina; EC, endothelial cell; PC, pericyte; RBC, red blood cell; TJ, tight junction.

IgG levels are maintained in human serum at around 10 mg/ml. The brain is isolated from serum by the blood-brain barrier (BBB), which is impermeable to large macromolecules including IgG (32). The brain, instead, is bathed in cerebrospinal fluid (CSF), which is produced following the filtration of blood and transport of ions across the choroid plexus. The resulting concentration of IgG in CSF is around 500- to 1,000-fold lower than in serum. At face value, this low concentration of antibody in the brain makes CNS antigens unattractive as targets for passive immunotherapy, which is normally administered to the periphery. This is compounded by a poor understanding of the mechanisms by which steady state levels of antibody are maintained. CSF flows around the brain, before exiting the CNS along spinal and cranial nerves and via drainage to the lymphatic system (33, 34). Intrathecally administered IgG is rapidly cleared from the brain, largely through this bulk flow and with a possible contribution of selective transport out of the brain. The neonatal Fc receptor, FcRn, is expressed in abundance at the BBB (35). Given FcRn's role in transcytosis of antibodies across the placenta, it has been suggested that FcRn may perform reverse transcytosis to help maintain the low IgG environment of the CNS. There is some evidence that antibody clearance from the brain is mediated in part by the antibody Fc domain (36, 37), and export of an anti-Aβ monoclonal antibody was reduced in an FcRn-deficient mouse (38). However, the brain concentration of peripherally administered IgG was not significantly different between wild-type mice and mice lacking FcRn (39). This speaks to a need for further investigation of how antibody levels in the CNS are maintained, with a particular requirement to understand the rate of transit across the BBB (Figure 2). Under a model where antibodies are maintained at static, low levels in the CNS, there is little scope for achieving meaningful binding occupancy to intracerebral antigens. However, if there exists a rapid cycling of antibodies in and out of the brain, total exposure of antibodies to antigen will, over time, be substantially greater. Evidence in support of a high flux dynamic equilibrium comes from experiments that measured the rate of clearance of intrathecally administered IgG, which demonstrated a half-life of <1 h in a primate model (40). This compares to a half-life of around 3 weeks in the periphery. Thus, peripherally produced or administered antibodies, particularly if they have high affinity for their antigens, may gain sufficient exposure to meaningfully engage intracerebral antigens.

The assertion that antibodies can bind antigens in the brain and exert biological effects is supported by several strands of clinical evidence. For instance, there is a growing set of neuroimmune diseases associated with auto-antibodies that bind to neuronal targets (41, 42). Antibodies against the membrane-associated protein amphiphysin are causally linked to the rare progressive disease stiff-person syndrome (43). Antibodies against antigens such as Zic4 and Yo/PCA1 are associated with cerebellar ataxia and antibodies against N-methyl-D-aspartate receptor (NMDAR) are commonly associated with encephalitis (44). From animal and clinical studies it has been shown that peripherally administered antibodies that target Aβ can engage their targets and induce reductions in brain amyloid load (45–49). Though an unwanted side effect, the ability of passive immunotherapy against Aβ to induce lesions (amyloid-related imaging abnormalities or ARIAs, discussed further below), stands as further testament to ability of antibodies to engage targets in the brain parenchyma. Together, these clinical observations stand as strong evidence that antibodies in circulation can penetrate the brain and engage their targets at a level sufficient to exert biologically relevant effects.

## Fc Receptors and Their Expression in the Brain

FcγRs are expressed on the surface of a wide range of immune effector cell types and bind to the Fc region of IgG. The canonical FcγRs are divided into those that activate immune signaling upon binding to antibody (in humans these are FcγRI, FcγRIIa, FcγRIIc, and FcγRIIIa), one that exerts inhibitory function (FcγRIIb), and one neutral glycosylphosphatidylinositol (GPI)-linked receptor, FcγRIIIa, which lacks cytoplasmic domains and is highly expressed on neutrophils (Table 1). There are four subclasses of IgG (IgG1, IgG2, IgG3, and IgG4) with varying affinity for the different receptors. The high-affinity interactions are between FcγRI and all IgG subclasses except IgG2, and between FcγRIIIa and IgG3 (50). The high-affinity interactions permit binding to free IgG molecules, yet are not of such high affinity that they preclude responses to IgG-labeled multivalent complexes (50). There is a widespread, but erroneous, belief that IgG4 is a neutral subclass of IgG. In fact, it binds all FcγRs, albeit with slightly lower affinity than IgG1 (50, 51). However, IgG4 does not fix complement and can inhibit IgG1-mediated complement fixation (52, 53). Uniquely among the human antibody subclasses, IgG4 undergoes arm exchange, resulting in chimeric, bispecific antibodies (54). In mice, the FcR system is broadly similar, with activating FcγRs (FcγRI, FcγRIII, and FcγRIV) and one with inhibitory activity (FcγRII) (Table 2). Like humans, there are four IgG subclasses, (IgG1, IgG2a, IgG2b, and IgG3) though the nomenclature differs between the species: for instance, IgG2a is most similar in its effector functions to human IgG1. The atypical Fc receptor TRIM21 is broadly expressed in the cytoplasm and possesses ubiquitin ligase activity. It can bind all classes of IgG (55) as well as IgA and IgM (56, 57). Following detection of intracellular immune complexes, TRIM21 stimulates a co-ordinated series of ubiquitination steps culminating in the degradation of immune complexes at the proteasome and an antiviral transcriptional response (56, 58–60).

**Table 1:** Human Fc receptors.

**Name**	**Activity**	**High affinity ligands**	**Low affinity ligands**	**Peripheral expression**	**Brain expression**
FcγRI	Activatory	IgG1, IgG3, IgG4		M**ϕ**, DC	MG
FcγRIIa	Activatory		All IgG subclasses	M**ϕ**, DC, Neutrophil, Basophil, MC, Eo, Pl	MG
FcγRIIb	Inhibitory		All IgG subclasses	B cells, Basophil, DC, M**ϕ**	MG
FcγRIIc	Activatory		All IgG subclasses	NK, M**ϕ**, Neutrophils	?
FcγRIIIa	Activatory	IgG3	IgG1, IgG2, IgG4	NK, M**ϕ**	MG
FcγRIIIb	Neutral		IgG1, IgG3	Neutrophils, Basophils	?
FcRn	Transcytosis, recycling	All IgG subclasses		M**ϕ**, DC, Neutrophil	BBB endothelium
TRIM21	Activatory/degradation	All IgG subclasses	IgA, IgM	Universal, high in M**ϕ**, DC, B cell	MG, neurons

*Summary of the localization of expression and binding characteristics of human cell surface FcγRs, the recycling Fc receptor, FcRn, and the cytoplasmic Fc receptor TRIM21. High-affinity interactions are defined as those with an dissociation constant (K_d_) <10^-7^ M. MΦ, monocyte/macrophage; DC, dendritic cell; Eo, eosinophil; NK, natural killer cell; MG, microglia; BBB, blood-brain barrier. Information for this table adapted from Bruhns and Jönsson (50), Vidarsson et al. (51), and McEwan (61). ? denotes that CNS expression is not defined*.

**Table 2:** Mouse Fc receptors.

**Name**	**Activity**	**High affinity ligands**	**Low affinity ligands**	**Peripheral expression**	**Brain expression**
FcγRI	Activatory	IgG2a	IgG2b	Mϕ, DC	MG
FcγRII	Inhibitory		IgG1, IgG2a, IgG2b, IgE	B cell, M**ϕ**, Neutrophil, DC	MG
FcγRIII	Activatory		IgG1, IgG2a, IgG2b, IgE	NK, M**ϕ**, Neutrophil, DC	MG
FcγRIV	Activatory	IgG2a, IgG2b	IgE	M**ϕ**, Neutrophil	MG
FcRn	Transcytosis, recycling	All IgG subclasses		Placenta, M**ϕ**, Neutrophil, DC	BBB endothelium
Trim21	Activatory / degradation	All IgG subclasses	IgA?, IgM?	Universal, high in M**ϕ**, DC, B cell	Neurons, MG

*Summary of the murine Fc receptors, their binding partners and pattern of expression. As in Table 1, high-affinity interactions are defined as those with an dissociation constant (K_d_)<10^-7^ M. MΦ, monocyte/macrophage; DC, dendritic cell; Eo, eosinophil; NK, natural killer cell; MG, microglia; BBB, blood-brain barrier; ? denotes possible interaction not yet demonstrated*.

The major site of Fc receptor expression in the brain is on the surface of microglia, the resident phagocytic immune effector cells of the CNS. In humans this includes the cell surface receptors FcγRI, FcγRIIa, FcγRIIb, and FcγRIIIa (62). There are reports of FcγRs on other cell types in the mouse brain, including on neurons (62, 63). However, other studies that sought evidence of FcγR expression at the transcript and protein level suggest that expression is minimal or absent in cells other than microglia (64, 65). The discrepancies between these findings may lie in the region of the brain analyzed as staining has been reported to be specific to regional neuron populations (66) or may reflect *ex vivo* vs. *in vivo* conditions. Outside the brain, FcγRI expression has been detected on sensory and motor neurons (67–69). TRIM21 is universally expressed and we have confirmed its expression in mouse primary neurons and human neuroblastoma cells (70). The neurodegenerating brain is an inflamed state, with widespread microglial activation and production of inflammatory cytokines including TNF, IL-6, and IL-1β (71, 72). Levels of TRIM21 and cell surface FcγRs are both increased following immune activation (58, 73). This is pertinent to the development of immunotherapeutics, as the degenerating brain may exhibit an exaggerated response to immune complexes. FcR upregulation may enhance the effectiveness of any Fc-mediated clearance mechanism, but has the potential to drive inappropriate immune stimulation. Trials of passively-transferred antibodies against Aβ have reported ARIAs, which are caused by intracerebral oedemas or microhaemorrhages (74). This represents a clear safety issue for immunotherapies and limits the range of doses available to clinicians. These adverse events are potentially driven by microglial activation following engagement of antibody-bound Aβ assemblies by FcγRs. Of note, studies on tau, αS and Aβ have reported mechanisms of protection that do not rely on engagement of cell surface FcγRs (65, 70, 75–79). Current clinical trials are therefore testing monoclonal antibodies with modified effector functions as a means to preserve activity whilst diminishing adverse events (80, 81). The nature of these immunotherapies will be discussed further below, before a discussion on their likely mechanisms of action.

## Clinical Immunotherapy

Several immunotherapies that target proteins implicated in neurodegeneration have entered human clinical trials. They fall into two categories: those that attempt to induce protective immunity in the patient through vaccination (active immunotherapy) or the infusion of monoclonal antibodies (passive immunotherapy). Active immunotherapy has as its benefit the sustained production of antibody from few vaccine doses. However, there remain issues of variable or incomplete protection between individuals and a risk that side effects may be long-lasting or irreversible. Passive transfer of monoclonal antibodies permits precise control of dosing and the epitope targeted, avoids stimulating a potentially damaging T-cell response and can be withdrawn in the event of adverse effects. However, the large quantities of recombinantly produced antibody that need to be periodically infused in passive immunotherapy approches come with considerable cost implications.

### Aβ Immunotherapies

The most advanced of the neurodegeneration immunotherapies are those that target Aβ (82), where 11 different approaches have reached clinical trials, seven of which are passively transferred monoclonal antibodies and four of which are active vaccination approaches (82) (Table 3). The first of these, AN1792, an active vaccine against full-length Aβ_42_, was halted following the occurrence of meningoencephalitis in 6% of the study population, all of whom had mild to moderate AD (83). Post-mortem analysis of two patients who developed meningoencephalitis indicated a T-cell mediated response was probably responsible for the inflammatory pathology. A second generation of active immunotherapies aims to target the N-terminus of the Aβ peptide, thereby avoiding a C-terminal T-cell epitope that may have been responsible for T-cell activation following vaccination with full-length Aβ (83). For the passive immunotherapies against Aβ, a reduction in Aβ PET biomarkers has been observed for gantenerumab, aducanumab, and bapineuzumab (46–48, 84). Safety issues mainly concern ARIAs, especially in carriers of the *APOE4* allele (85). ARIAs are likely due to the antibody decoration of Aβ plaques and the use of antibodies that preferentially bind Aβ monomers over fibrils (e.g., solanezumab) may therefore represent a mechanism to avoid them. Despite the evidence of target engagement, there is no evidence of clinical benefits for any of the drugs that have been tested in Phase III trails, which are powered to test efficacy. As the Phase III trials conducted to date have been conducted in patient groups with established AD, their failure suggest earlier treatment may be critical for cognitive benefits. Future trials will test this hypothesis in populations with dominantly inherited dementias, or at risk of developing sporadic AD based on PET Aβ accumulation, using gantenerumab and solanezumab (86, 87). There therefore remains cause for hope in the targeting of Aβ in AD, but, if it is to be successful, it will likely require early intervention, a pre-requisite of which is predictive diagnostics.

**Table 3:** Clinical immunotherapies in neurodegeneration.

**Name**	**Immunotherapy type**	**Target**	**Company**	**Most advanced clinical trial ID**	**Phase of trial**
**IMMUNOTHERAPIES TARGETING** **αS and TAU**					
AADvac1	Active	Tau 294–305	Axon Neuroscience	NCT02579252 (mild AD)	Phase II
					
ACI-35	Active	Tau pS396, pS404	AC Immune & Janssen	ISRCTN13033912 (mild to moderate AD)	Phase Ib
					
BIIB054	Passive	α-synuclein	Biogen, Neurimmune	NCT03318523 (PD)	Phase II
BIIB076	Passive, huIgG1	Tau	Biogen, Neurimmune	NCT03056729	Phase I
BIIB092	Passive, huIgG4	Tau N-terminus	Biogen & Bristol-Myers Squibb	NCT03068468 (PSP)	Phase II
					
				NCT03352557 (early AD)	Phase II
C2N-8E12	Passive, huIgG4	Tau 25-30	AbbVie & C2N Diagnostics	NCT02985879 (PSP)	Phase II
				NCT02880956 (early AD)	Phase II
PRX002	Passive, huIgG1	α-synuclein 118-126	Hoffmann La Roche, Prothena	NCT03100149 (early PD)	Phase II
RG7345	Passive	Tau pS422	Hoffmann La Roche	NCT02281786	Phase I (discontinued)
RO7105705	Passive, huIgG4	Tau	AC Immune SA, Genentech & Hoffmann La Roche	NCT03289143 (prodromal to mild AD)	Phase II
				NCT03828747 (moderate AD)	Phase II
LY3303560	Passive	Tau conformational epitope	Eli Lilly	NCT03518073 (early AD)	Phase II
JNJ-63733657	Passive	Tau mid-region	Janssen	NCT03375697	Phase I
UCB0107	Passive	Tau 235–246	UCB	NCT03464227	Phase I
**SELECTED IMMUNOTHERAPIES TARGETING Aβ**					
Solanezumab	Passive IgG1	Aβ (monomeric)	Eli Lilly	NCT02008357 (at risk of AD / mild AD)	Phase III
				NCT01760005 (fAD)	Phase III
Gantenerumab	Passive IgG1	Aβ (assembled)	Chugai Pharmaceutical, Hoffmann La Roche	NCT01760005 (fAD)	Phase III
				NCT03444870 (early AD)	Phase III
AN1792	Active	Aβ42	Pfizer, Janssen	NCT00021723	Phase II (terminated)
Aducanumab	Passive IgG1	Aβ (assembled)	Biogen, Neurimmune	NCT02484547 (early AD)	Phase III
Bapineuzumab	Passive IgG1	Aβ (assembled and soluble)	Pfizer, Janssen	NCT00998764	Phase III (terminated)

*A summary of immunotherapies against tau and αS that have entered, or are soon to enter, clinical trials and selected immunotherapies against Aβ. PSP, progressive supranuclear palsy; AD, Alzheimer's disease; PD, Parkinson's disease; fAD, familial Alzheimer's disease*.

### Immunotherapy Against Cytoplasmic Proteins

Over the past decade, it has been repeatedly shown that active vaccination against tau or αS can alleviate the burden of pathology in the mouse brain (88–94). The mechanism of this immune protection is likely mediated by humoral immunity, as passive transfer of anti-tau antibodies is sufficient to confer a protective effect (95–100). This situation is reminiscent of viral infections, where the passive transfer of antibodies often confers sterile protection against infection (101, 102). Encouraged by the reductions in protein pathology, preservation of brain volume and ameliorations of behavioral metrics in mouse studies, clinical trials of tau and αS immunotherapies have commenced, or are planned (Table 3) (103, 104). We here summarize the therapies, and the rationale behind them, using the available pre-clinical and clinical data.

### AADVac1

Following screening of antibodies that inhibited *in vitro* aggregation of recombinant tau, a monoclonal antibody, DC8E8, was identified with potent inhibitory activity (105). The epitope of this antibody is HXPGGG, a motif present in each of the four repeat domains of full-length tau. Passive transfer of DC8E8 was protective in a transgenic mouse expressing truncated human tau. This data was used to select an epitope for active vaccination (tau 294-305 KDNIKHVPGGGS), conjugated to keyhole limpet hemocyanin (KLH). In transgenic rats expressing truncated human tau, the vaccine was alum-adjuvanted and was found to confer a reduction in total and hyperphosphorylated tau species (106). Following these findings, human trials of the vaccine were commenced. A Phase I trial demonstrated that vaccination successfully induced an anti-tau immune response in 29/30 patients, which was biased toward an IgG1 response (107, 108). Phase II trials are underway in mild AD and primary progressive aphasia patients (109).

### ACI-35

ACI-35 is a 16mer peptide comprising tau residues 393–408 with phosphorylation at S396 and S404 (91). This overlaps with the epitope of PHF1, an antibody widely used to detect pathological tau species (110). In the ACI-35 vaccine, the doubly-phosphorylated tau peptide is delivered in liposomes. Vaccination conferred a reduction in levels of soluble and insoluble tau phosphorylated at S396 in mice transgenic for human P301L tau (91). Protection against other phosphorylation sites of tau were not observed. Levels of insoluble tau were reduced but were not statistically significant by conventional criteria. The vaccine promoted the rescue of a clasping defect in the P301L tau transgenic mice but had no effect in the Rotarod test, a more demanding agility task. From a safety perspective, there was no observed influx of lymphocytes and no induction of astrogliosis. A Phase Ib clinical trial is currently taking place in AD patients with mild to moderate symptoms.

### BIIB076

BIIB076 is a fully human IgG1, derived from Neurimmune's reverse translational approach, which mines antibody sequences isolated from humans. Little pre-clinical work has been published for BIIB076, though it was found to bind with subnanomolar affinity to human and cynomolgus macaque tau (111). When given to macaques it was found to reduce total and unbound CSF tau at the highest doses. A Phase I trial is under way.

### BIIB092

Induced pluripotent stem cell-derived neurons prepared from familial AD patients secrete a series of truncated tau products that were termed eTau (112). eTau species consist of the N-terminal region of tau and run between 20 and 35 kDa by SDS-PAGE western blot. When added to primary cortical neurons, eTau caused neuronal hyperactivity and upregulated the expression of Aβ. The authors propose a model wherein secreted tau creates a destructive feed forward loop: Aβ drives tau pathology and secreted tau in turn upregulates Aβ. The antibody IPN002 binds the N-terminus of tau and neutralizes the effect of eTau. *In vivo*, it reduced levels of tau in CSF in P301L tau transgenic mice. IPN007, the humanized IgG4 version of the antibody, now renamed BIIB092, is being evaluated in Phase II trials in PSP and early AD patient populations.

### C2N-8E12

C2N-8E12 is a humanized IgG4 version of the antibody HJ8.5 that binds with picomolar affinity to the N-terminal region of tau at residues 25–30 (95). In a seeding assay in human embryonic kidney cells (113), HJ8.5 exerted potent protection against tau seeds isolated from aged mice expressing human P301S tau (95). When chronically perfused into the ventricles, or delivered intraperitoneally to the same mouse model, HJ8.5 substantially reduced the extent of staining with antibodies specific for pathological tau and improved cognition (95, 96). Two Phase II clinical trials of C2N-8E12 are currently in progress in PSP and early AD cohorts.

### RG7345

RG7345 is a humanized rabbit monoclonal that targets a C-terminal epitope of tau phosphorylated at S422 (100). The antibody was found to specifically enter neurons that contained hyperphosphorylated tau, suggestive of a target-dependent uptake mechanism. When injected to the periphery of TauPS2APP mice, which express human P301L tau and mutant forms of APP and PSEN2, the antibody reduced levels of tau phosphorylated at S422. The drug entered a Phase I clinical trial that was completed in 2015. However, no results have been posted for the trial and Roche discontinued development for unknown reasons.

### RO7105705

Genentech and AC Immune published work demonstrating that a mouse IgG2b antibody that targets tau phosphorylated at S409 reduced pathological tau staining in P301L tau transgenic mice (65). Mutation of the D265A and N297G (DANG) residues of the antibody, which prevents binding of cell surface FcγRs but not to murine complement (114), did not substantially reduce protection but prevented release of inflammatory cytokines by microglia (65). This work likely informed the selection of the IgG4 backbone for RO7105705, which has reduced capacity to engage microglia when compared to other subclasses (115). The precise epitope of RO7105705 has not been disclosed though it was reported to target the N-terminus (116). Two Phase II trials in prodromal to mild AD and moderate AD cohorts are in progress.

### JNJ-63733657 and UCB0107

Based on the rationale that antibodies targeting the termini of tau may ineffectively bind proteolytically-digested tau assemblies, Janssen and UCB have selected antibodies that target the mid-domain of tau. The antibodies both reportedly block seeded aggregation of tau in cell-based seeding assays (117). These antibodies both recently entered Phase I clinical trials.

### LY3303560

Eli Lilly have humanized a well-characterized conformation-specific antibody, MC-1 (118), which binds a discontinuous epitope of tau comprised of the N-terminal EFE motif (residues 7–9) and the core region (residues 313–322). From cryo-electron microscopy structures, the EFE motif is hypothesized to interact with the core structure in the AD paired-helical and straight filaments (119). Accordingly, LY3303560 displays a preference for binding aggregated over monomeric tau (120). Little preclinical data have been published and the antibody subclass has not been disclosed. A Phase II clinical trial is currently under way in sufferers of early symptomatic AD.

### PRX002

PRX002 is being developed by Roche and Prothena as an αS-targeting immunotherapy. The mouse monoclonal, 9E4, from which PRX002 derives, belongs to the IgG1 subclass (121). 9E4 targets a C-terminal epitope of αS (residues 118–126), with a preference of monomeric over assembled versions of αS (122). When repetitively delivered intraperitoneally to a mouse model that over-expresses wildtype human αS, 9E4 protected against neuronal cell loss and improved behavioral parameters including Rotarod (121). Levels of a C-terminal fragment of αS, and higher-order assemblies of αS, were reduced by passive vaccination. 9E4 was found to accumulate in neurons and co-localize with αS and with lysosomal (cathepsin D) and autophagosomal (LC3) markers. Consistent with induction of αS degradation via autophagy as 9E4's mechanism of action, the intensity of LC3 staining was increased following 9E4 treatment, and clearance of αS by 9E4 was prevented by inhibition of autophagy in neuronal cultures. Phase I trials reported favorable safety and pharmacokinetics with evidence of target engagement in serum (123). A Phase II trial is currently in progress in early PD patients.

## Mechanisms of Antibody-Mediated Protection

Numerous mechanisms have been posited for how antibodies may deplete aggregated proteins in the brain. As with protection against viral infection, particular mechanisms likely dominate the activities of individual monoclonal antibodies. The selection of individual monoclonal antibodies for passive immunotherapy, and modulating antibody effector function, is therefore a means by which specific effector functions may be selected. We will here delineate the mechanisms that are likely to operate against protein aggregates and, where possible, relate them to pre-clinical and therapeutic studies.

### Peripheral Sink

The peripheral sink hypothesis posits that antibodies binding to targets in the periphery will shift equilibrium dynamics across the BBB, thereby reducing the concentration of cerebral antigens (124). This provides a mechanism that would enable the depletion of cerebral antigens by administering antibodies that promote clearance in the periphery. If biologically valid, it would enable the problem of antibody penetrance to the brain to be bypassed, as maintaining peripheral pools would be sufficient to promote CNS antigen depletion. The application of the peripheral sink hypothesis has been mostly applied to Aβ, which can be readily detected in both CSF and serum and therefore potentially susceptible to this process. Solanezumab and ponezumab both selectively target monomeric Aβ, and have been proposed to operate via the peripheral sink mechanism (125, 126). Consistent with a transfer to periphery, both antibodies, as well as a monomer-binding monoclonal antibody, m266 which was given to mice, increased the serum concentration of Aβ (125, 127, 128). However, this may be an effect of prolonging the half-life of Aβ in serum by complexing with antibody, as concentrations of free Aβ were not diminished. In a non-human primate model, the enzymatic degradation of Aβ in the periphery, although efficient, did not reduce CNS Aβ load (129). This experiment implies that degradation of CNS antigens in the periphery is not sufficient to substantially reduce CNS load. However, it must be noted that anti-Aβ IgG may itself alter the rate of efflux of antigen from the brain by promoting export of antibody:antigen complexes and the enzymatic degradation experiment would not capture this effect. Local production of antibodies against proteopathic agents within the brain, or administration of antibodies directly to the CNS, is associated with high levels of protection (95, 130–133). This demonstrates that mechanisms that rely on direct CNS exposure to antibodies can dominate protective effects. It is therefore likely that in order to target CNS antigens effectively, the problem of low antibody concentration in the brain must be confronted head-on by ensuring sufficient intracerebral target engagement for therapeutic efficacy.

### Neutralization

It has been known since the 1930s that the incubation of virus particles with antibodies often results in a reduction in infectious titer, a phenomenon termed neutralization (134). Given the mechanistic similarities between viruses and cytoplasmically replicating proteopathies such as tau and αS, there is value in comparing the effects of antibody on both types of pathogen. Neutralization can only effectively be studied in cell-based models, outside a living organism, as the professional immune system confounds observations. We will here extend established definitions of virus neutralization (135) to cytoplasmic seeded protein misfolding as:

*the reduction in seeding potency observed following the binding of antibodies to proteinaceous assemblies in cell-based seeding or propagation assays in the absence of complement or cells of the professional immune system*.

This definition therefore excludes the effects of microglial clearance and other effector mechanisms that are likely to operate *in vivo*. The cellular substrate used for examining the effect of antibodies on seeding ability is typically mouse primary neurons, human cell lines, or, more recently, human neurons derived from induced pluripotent stem cells (136, 137). It remains to be determined whether the choice of cellular model influences the extent, and mechanism, of observed neutralization.

Antibodies that exert potent neutralizing responses against viruses in cell based systems frequently exert strong *in vivo* protection (135, 138, 139). Indeed, a neutralizing antibody response is considered a surrogate marker of protective immunity in many circumstances. Until recently neutralization was thought to be synonymous with preventing entry of viruses, or, more specifically, their genomes, to the interior of the cell (135). A post-entry mechanism of neutralization that relies on engagement of the intracellular Fc receptor TRIM21 has recently been characterized, and is discussed further below. For entry-blocking antibodies, though the end result is identical (viruses fail to enter the cell), there are numerous mechanisms by which this may be achieved. For example, antibodies may effect a block to entry by preventing engagement of cell surface receptors, agglutinating virus particles or blocking escape from endosomes, each of which ultimately results in a block to virus entry.

**Figure 3:**
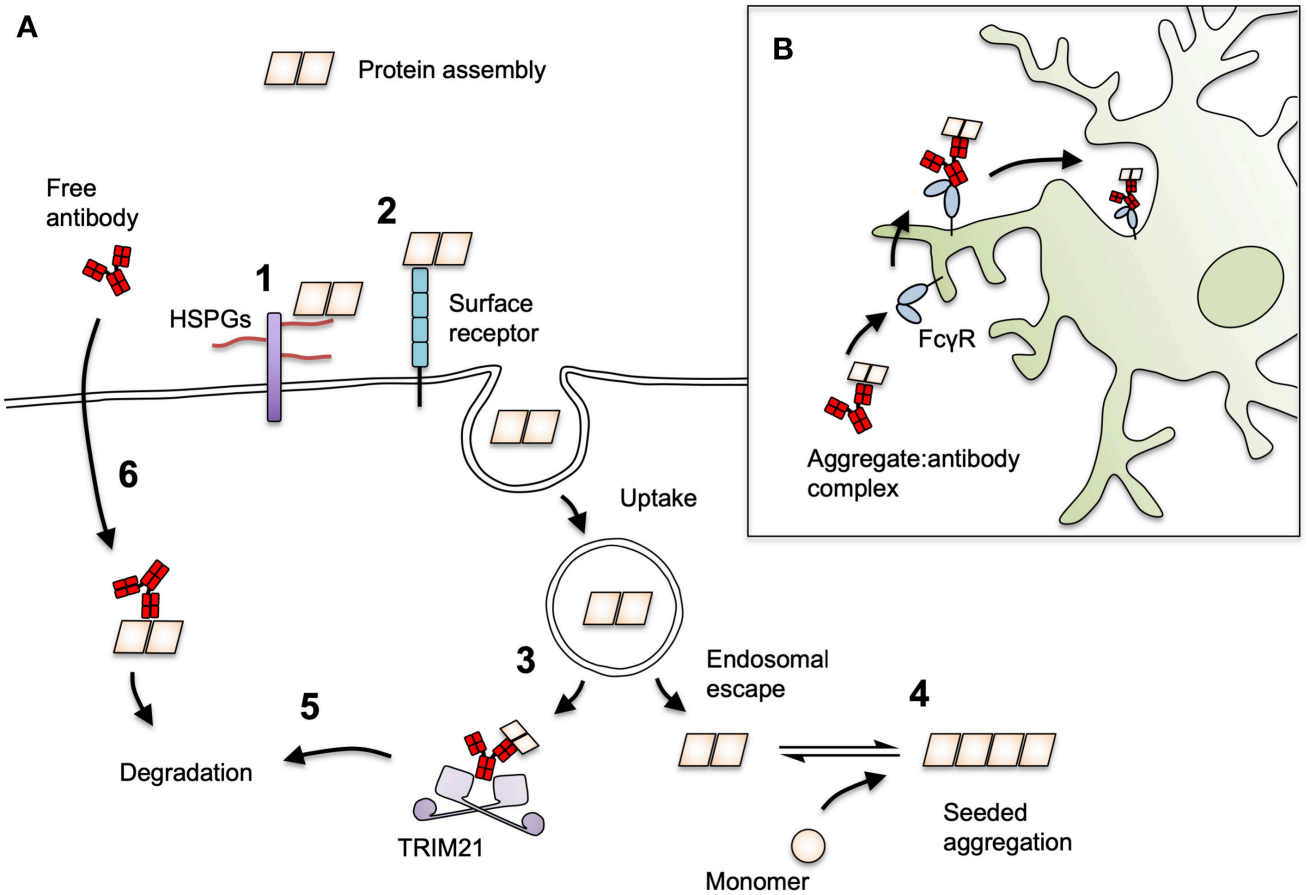
Mechanisms of antibody-mediated protection against prion-like proteins. **(A)** The process of seeding for tau and αS may be neutralized by antibodies at several stages. Protein seeds attach to cells via interactions with (1) heparan sulfate proteoglycan (HSPGs) or (2) cell surface receptors such as LAG3 for αS. (3) Seeds must escape vesicular compartments in order to induce seeding, a step that by analogy with viral infection could be inhibited by antibodies. (4) Seeds that escape to the cytoplasm with antibodies attached may be prevented from undergoing seeded aggregation or (5) become targets for proteasomal destruction by the cytoplasmic Fc receptor and ubiquitin ligase, TRIM21. (6) Antibodies may be directly taken up into cells in a target-specific manner and mediate degradation of target proteins in the cytoplasm via TRIM21, or in the lysosome/autophagy pathways. **(B)** Antibody-decorated aggregates can be ligated by cell surface FcγRs on microglia. This induces their uptake and degradation and may play an important role in overall *in vivo* protection.

In proteopathic seeding experiments, antibodies have been documented to reduce or slow the uptake of tau to cells. Examples of these are the anti-tau monoclonal antibody HJ9.3 (79) and a polyclonal preparation against the tau C-terminus, which slowed the uptake of tau to iPSC-derived neurons (136). Likewise, the anti-αS antibodies Syn211 and Syn303 reduced the uptake of αS fibrils to mouse hippocampal neurons (78). Together, these findings demonstrate that entry blocking neutralization can operate against protein assemblies (Figure 3A). However, entry-blocking is by no means a universal mechanism, since the antibody HJ8.5, which potently neutralizes seeding (94), fails to block tau uptake to neurons (79). The N-terminal monoclonal antibody 5A6 (140) and a C-terminal polyclonal, BR134 (141), similarly exert neutralization activity without substantially preventing uptake (70). For these latter two antibodies, neutralization activity relies on intracellular neutralization via TRIM21. Further, without a firm understanding of the mechanisms of seed entry to the cell, it is not clear exactly how antibodies elicit a block to cellular uptake. For αS fibrils, interactions with the putative entry receptor LAG3 facilitate binding and uptake to cells (142). Inhibition of this interaction with anti-LAG3 antibodies C9B7W and 410C9 reduced αS uptake. For both αS and tau, interactions with sulfated proteoglycans promote aggregate uptake (143–145) and inhibition of this interaction is the proposed mechanism for HJ9.3 (79).

By analogy with viruses, it is conceptually possible that antibodies block entry to the cytosol at a post-uptake stage, for instance by blocking endosomal escape, or by promoting endolysosomal degradation. There has been little study on the ability of antibodies to act at a post-uptake, pre-cytosolic entry stage. Implementation of the necessary methods is technically challenging, and, as with approaches for viral infection, particles that have escaped to the cytoplasm must be reliably differentiated from the endosomal population, which is likely to be overwhelmingly greater. Surrogate markers of tau and αS entry to the cytoplasm, such as Galectin 3-GFP, which binds carbohydrates on disrupted endosomes, have been developed (146, 147) and could be usefully applied to the field of antibody neutralization.

For certain non-enveloped viruses, neutralization can occur entirely independently of entry blocking. Antibodies against adenovirus do not prevent entry to the cell but remain associated with viral particles in the cytoplasm. Once in the cell, antibodies are bound by the cytoplasmic, high-affinity Fc receptor TRIM21, which mounts a rapid degradation response against the immune complex (Figure 3A). This substantially reduces viral infectivity and genetic deletion of TRIM21 renders certain antibodies non-neutralizing (56, 60). Mice that lack TRIM21 are highly susceptible to viral infection and, unlike their wild-type counterparts, cannot be fully protected by passive transfer of neutralizing antibodies (148). The distinguishing feature of viruses that are susceptible to TRIM21 is that their capsids are naked (i.e., without lipid bilayer) and lack fusogenic or membrane pore-forming mechanisms that permit the separation of genomic material and antibody-bound antigens during entry. Rather, these TRIM21-sensitive viruses, which include adenoviruses and minor group rhinovirus (149), enter the cell through lysis of the endosome, leaving the antibody-bound virus particle exposed (150, 151). The uptake of naked protein assemblies and entry to the cytosol though spontaneous or aggregate-induced lysis of vesicles (146, 147), is, similarly, a route that allows access of antibodies to the cytoplasm. Indeed, several studies have found that antibodies are taken up with exogenously added tau seed, and that antibodies do not prevent tau uptake to neurons (70, 79, 96, 136). Antibody-coated tau assemblies that escape to the cytoplasm become associated with TRIM21, and are prevented from inducing seeded aggregation by its activity (70). The extent to which intracellular neutralization by TRIM21 contributes to the overall *in vivo* protection afforded by an antibody remains to be determined.

### Clearance by Microglia

Microglia display an ability to take up naked assemblies of tau and αS and induce their degradation (152, 153). When in complex with antibodies, cellular uptake and degradation of both tau and αS is enhanced (79, 154, 155). This activity is Fc-dependent, as use of F(ab')2 fragments, which lack the Fc domain, or FcγR blocking antibodies, prevent clearance. This represents a mechanism that can be exploited for the therapeutic clearance of protein deposits (Figure 3B). However, FcγR-mediated clearance of protein deposits comes with a risk of activating a damaging immune response, as likely occurred during immunotherapy that targeted Aβ plaques (74, 85). Several immunotherapies have selected IgG4 as a scaffold with a rationale that it may minimize damaging pro-inflammatory responses (Table 3). However, as noted above, IgG4 binds FcγRs (50) and any reduction in inflammatory induction by IgG4 may owe more to its inability to fix complement (52, 53). Nonetheless a side-by-side comparison of an anti-Aβ antibody, MABT, with human IgG1 vs. IgG4 constant regions demonstrated a reduced ability of IgG4 to promote microglial inflammation by Aβ:antibody immune complexes (115). Two recent clinical trials with anti-Aβ IgG4 antibodies with reportedly low ability to engage FcγRs have commenced (80, 81). Uncertainty therefore persists in the selection of antibody isotypes for immunotherapy for maximal therapeutic effect and the extent to which isotype selection influences effector function in the brain. Passive immunotherapies on human IgG1 (BIIB076, PRX002) and IgG4 (BIIB092, C2N-8E12, RO7105705) scaffolds have been selected for clinical trials. Though an imperfect experiment, results of Phase II and III clinical trials, when considered together, will hopefully provide insight regarding the effect of isotype selection on therapeutic outcomes.

As noted above, an anti-pS409 tau antibody that possesses the DANG point mutations that prevent FcγR engagement retains the ability to prevent tau spread and neurotoxicity (65). Thus for antibodies that confer protection via alternative mechanisms, dispensing with FcγR engagement altogether provides a potential safety advantage. Other studies have reported that an antibody against pS404 of the mouse IgG2a isotype, which preferentially binds to activatory FcγRs (50), was more potent at clearing tau pathology than a mouse IgG1, which possess enhanced binding to the inhibitory FcγRII, despite targeting the same epitope with similar affinity (97). This would suggest that activatory microglial engagement, at least for these antibodies, has a net protective effect. Indeed, it has been argued that microglial engagement is both well-tolerated and therapeutically desirable (108). To satisfactorily address these issues, future work should determine the effect of antibody subclass on levels of *in vivo* protection by isotype switching monoclonal antibodies.

### Intracellular Sequestration or Clearance

Free antibodies against tau have been found to enter neurons in cell based systems and in passive transfer experiments in mice (89, 99, 100, 156). Antibodies were found in complex with tau in the endolysosomal/macroautophagy pathways, suggesting that degradation is stimulated by antibody uptake. Antibody uptake could be blocked with antibodies against FcγRII/III in mouse neurons (99). The extent of this phenomenon is not clear, especially given the ambiguity concerning FcγR expression on the surface of neurons. The humanized antibody, MAb86/RG7345, was reported to enter neurons and was found associated with lipid rafts and intracellular or vesicular tau deposits (100). However, clinical trials for this antibody were discontinued for reasons that have not been disclosed. Intracellular sequestration is therefore a mechanism of action that is not explicitly represented in current clinical trials that target tau. It remains to be determined whether the phenomenon of intracellular antibodies involves the wholesale transfer of antibodies to the cytoplasm, or whether vesicles containing tau and antibody meet without cytoplasmic access. In the case of the former, it is expected that intracellular antibodies would be rapidly bound by TRIM21. It is therefore interesting that a monoclonal antibody, cis-113, specific to a soluble *cis*-tau conformer, was taken up by neurons and found to induce intracellular degradation of tau that was dependent on TRIM21 (157). Thus, both import of antibody in complex with tau seeds and uptake of free antibody by neurons may enable intracellular degradation of pathological protein species via the TRIM21 pathway (70, 157). Recent work demonstrates that TRIM21 can rapidly degrade diverse cellular proteins in experimental systems (158). It may therefore be possible to use antibodies and TRIM21 to specifically target disease-relevant protein conformations for degradation in the cytoplasm.

## Concluding Remarks

The evidence that protein aggregation spreads in a prion-like manner is accumulating and compelling. The immune system is tasked with the detection and destruction of pathogens. In the case of tau, αS and other protein agents, these tasks are evidently not performed to a sufficient degree to resolve or limit aggregation. Notably, the detection of aggregated proteins as a threat is hindered by a lack of classical pathogen-associated molecular patterns, arising from their status as self proteins in an altered conformation. Destruction is hindered due to their physically robust and highly compacted nature, which is refractory to proteasomal degradation (159). Antibodies, either induced following active immunization or passively transferred, represent a means by which protein assemblies can be labeled as threats and then inactivated by neutralization, sequestration, or FcR-mediated effector functions. A deeper understanding of these mechanisms may provide a route to novel therapies in age-related neurodegenerative disease. Finally, the expense of long-term passive immunotherapy may ultimately prove prohibitive to its widespread clinical uptake. However, evidence of improvement in cognitive outcomes following immunotherapy would serve as a critical indicator that pathologically important processes have been targeted. In this way, passive immunotherapy may serve as proof-of-principle for future therapies, targeting the same processes, that are more suited to scaled production at affordable cost.

## Author Contributions

WM wrote the manuscript. TK, BT, AM, and WM prepared figures and edited the manuscript.

### Conflict of Interest Statement

The authors declare that the research was conducted in the absence of any commercial or financial relationships that could be construed as a potential conflict of interest.
